# Virtual care in Ontario community health centres: a cross-sectional study to understand changes in care delivery

**DOI:** 10.3399/BJGPO.2021.0239

**Published:** 2022-06-01

**Authors:** Sara Bhatti, Simone Dahrouge, Laura Muldoon, Jennifer Rayner

**Affiliations:** 1 Alliance for Healthier Communities, Toronto, Canada; 2 Bruyère Research Institute, University of Ottawa, Ottawa, Ontario, Canada; 3 Family Physician, Somerset West Community Health Centre, Ottawa, Ontario, Canada; 4 Department of Family Medicine, University of Ottawa, Ottawa, Ontario, Canada; 5 Centre for Studies in Family Medicine, Western University, London, Canada

**Keywords:** primary health care, virtual care, telemedicine, COVID-19

## Abstract

**Background:**

There has been a large-scale adoption of virtual delivery of primary care as a result of the COVID-19 pandemic.

**Aim:**

In this descriptive study, an equity lens is used to explore the impact of transitioning to greater use of virtual care in community health centres (CHCs) across Ontario, Canada.

**Design & setting:**

A cross-sectional survey was administered and electronic medical record (EMR) data were extracted from 36 CHCs.

**Method:**

The survey captured CHCs‘ experiences with the increased adoption of virtual care. A longitudinal analysis of the EMR data was conducted to evaluate changes in health service delivery. EMR data were extracted monthly for a period of time before the pandemic (April 2019–February 2020) and during (April 2020–February 2021).

**Results:**

In comparison with the pre-pandemic period, CHCs experienced a moderate decline in visits made (11%), patients seen (9%), issues addressed (9%), and services provided (15%). During the pandemic period, an average of 54% of visits were conducted virtually, with telephone as the leading virtual modality (96%). Drops in service types ranged from 28%–82%. The distribution of virtual modalities varied according to the provider type. Access to in-person and virtual care did not vary across patient characteristics.

**Conclusion:**

The results demonstrate a large shift towards virtual delivery while maintaining in-person care. No meaningful differences were found in virtual versus in-person care related to patient characteristics or rurality of centres. Future studies are needed to explore how to best select the appropriate modality for patients and service types.

## How this fits

The COVID-19 pandemic resulted in a massive shift towards virtual delivery of primary health care. Published studies have reported changes in healthcare visits and services; however, this study reports changes in visit modality (that is, in-person, telephone, video, and text or email) along with changes in the number of healthcare visits, services provided, and issues addressed. The study also uses an equity lens to examine the distribution of visit modalities across sociodemographic factors (for example, sex, income, and education) of patients. This article adds to the existing literature on the impact of the COVID-19 pandemic to healthcare service delivery and will be helpful for primary care organisations working towards providing more equitable care.

## Introduction

Virtual delivery of primary care is not an innovation. In fact an article in an 1879 medical journal mentions the use of telephone visits as a means to reducing office visits.^1^ However, only recently has there been widespread adoption of virtual care owing to the COVID-19 pandemic. Before the pandemic, many barriers prevented greater implementation and use. For example, according to the Canadian Medical Association, the most notable challenge preventing greater uptake has been the absence of billing codes relevant to virtual care and reimbursement issues.^2^ Other commonly cited challenges include provider and patient readiness, worsening inequitable access to technology and resources, low digital literacy, concerns regarding the security and privacy of information, and quality of care concerns (for example, uncertainty on how to triage patients for certain modalities of care).^3–8^

These concerns, especially those related to exacerbating inequitable access, were shared by the Ontario CHCs during the rapid adoption of virtual care. CHCs are comprehensive, interprofessional, salary-based primary healthcare organisations with a long history of serving marginalised communities and addressing social determinants of health. CHC priority populations include people living in poverty, those in rural and remote areas, as well as those facing other barriers to access such as newcomers and people experiencing homelessness.^9–11^ With the advent of the COVID-19 pandemic and government-mandated physical distancing, primary care practices, including CHCs, were required to rapidly shift and offer virtual delivery of care.^12^ A number of studies have demonstrated that this accelerated transition to virtual care led to changes in healthcare visits and visit modalities using electronic medical record and billing data.^13–16^ Yet, currently, there is limited literature on the distribution of specific virtual modalities of care for issues addressed, services provided, provider types, and patient sociodemographic characteristics. In this descriptive study, an equity lens is used to explore the impact of transitioning to greater use of virtual delivery of primary care in CHCs.

## Method

### Study design and setting

A cross-sectional survey of CHCs was conducted to capture the organisations’ experiences with the increased use of virtual delivery of care during the COVID-19 pandemic, and a longitudinal analysis of EMR data to evaluate changes in health service delivery over time.

### Study population

All 73 CHCs operating in Ontario were eligible for the study. An invitation to participate was sent to all CHCs in May 2020. Only patients who had at least one CHC visit during the 22 months‘ study span were analysed.

### Data sources and collection

An e-survey link to the organisational survey was sent to clinical directors at each participating centre on 6 July 2020, and it was requested to be completed within 3 weeks. The eight-page survey was created based on existing literature on virtual care at the time of the study. The electronic survey captured information regarding rurality, staffing structure, which primary care services were performed virtually (that is, telephone, video, and text or email), how centres transitioned services for virtual delivery, as well as implementation challenges incurred (see Supplementary Appendix S1).

All Ontario CHCs use a common EMR and the same reporting data standards. Each centre’s EMR is extracted nightly to a centralised data warehouse where it is validated. From this data warehouse, the following were extracted: patient sociodemographic information; encounter information including visit modality (that is, in-person, telephone, video, and text or email); services provided; issues addressed; and provider type. All of this information was inputted directly into the EMR by providers. ‘Issues addressed’ refers to the specific health concerns including those related to the determinants of health that were addressed during the visit. Provider types included physicians, nurse practitioners, nurses, chiropodists, counsellors, dieticians, and diabetes educators. EMR data were extracted monthly for a period of time before the pandemic period (1 April 2019–28 February 2020) and during (1 April 2020–28 February 2021).

### Statistical analysis

EMR data were analysed at the practice level, and the monthly mean and standard deviation were reported as well as median and interquartile range for the total number of visits, virtual visits, unique patients seen, issues addressed, and services provided. The issues addressed were grouped into 12 broad clinically meaningful categories, which were validated by a CHC clinician and the research team (see Supplementary Table S1). The distribution of each visit modality type for all 12 categories was calculated for the COVID-19 time period only. The percentage change for each service type across the two time periods was calculated. Distribution of modalities was calculated for each provider type and sociodemographic variable (for example, age, sex, education, preferred language of service, household income, and household composition). The data stratified by rurality were also analysed to observe differences between urban and rural contexts.

## Results

### CHC characteristics

Thirty-six (49%) CHCs consented to the study, which were generally comparable with CHCs in Ontario with respect to geography, populations served, and size. Participating organisations had on average 28 full-time equivalent staff and 8897 registered patients, and most (61%) were located in urban areas (see Table 1). Patients were primarily female (58%), aged >61 years (35%), and more than one-quarter had completed high school education or less.

**Table 1.** Community health centres and patient characteristics

Community health centres (*n* = 36)^a^	*n* (%)^b^
Average number of registered patients, mean (SD)	8897 (5035)
Size of practice, average FTE for all staff, mean (SD)	28 (17)
Practice geography, urban	22 (61)
**Patient characteristics (*n* = 173 503)^c^**	
Age, years	
0–12	14 616 (8)
13–26	21 239 (12)
27–40	29 985 (17)
41–60	47 606 (27)
>61	60 057 (35)
Sex, female	99 990 (58)
English as preferred language of service	124 547 (72)
Education, high school or less	44 651 (26)
Household income	
$0–$24 999	37 343 (22)
$25 000–$59 999	22 615 (13)
>$60 000	16 314 (9)
Unknown	97 231 (56)
Living alone	22 316 (13)

^a^Data source: organisational survey. ^b^Unless otherwise stated. ^c^Data source: electronic medical record. FTE = full-time equivalent. SD = standard deviation.

Most practices reported they felt reasonably prepared to offer virtual delivery for primary care (*n* = 27, 75%) and health promotion (*n* = 22, 61%) services. Common challenges reported related to availability of resources required to deliver and receive virtual services (*n* = 17, 47%), and the patients’ digital literacy (*n* = 14, 39%). Centres reported concerns specifically for those living in poverty, seniors, newcomers, those experiencing homelessness, and people with mental health problems and addictions (data not shown).

### Transition to virtual care

In comparison with the pre-pandemic time period, the number of activities taken place monthly over the study‘s 11-month period declined (Table 2). The average monthly number of visits, unique patients seen, issues addressed, and services provided dropped between 9% and 15%.

**Table 2.** Median and mean numbers of visits, patients, issues addressed, and services provided during April 2019–February 2020 (pre-COVID-19) and April 2020–February 2021 (COVID-19)

Variable	Pre-COVID-19 (*n* = 396)		COVID-19 (*n* = 396)		Mean % change	Median % change
	Mean (SD)	Median (IQR)	Mean (SD)	Median (IQR)		
Total visits	2901.6 (1447)	2721.5 (1764–3592)	2572 (1482)	2145 (1510–3209)	11	–21
Total patients seen	480.7 (307)	429.5 (252–617)	438.8 (257)	396 (259–552)	9	–8
Total issues addressed	6349.3 (3930)	5697 (3940–7176)	5771 (4729)	4687 (3406–6553)	9	–18
Total services provided	8976.3 (5574)	7662.5 (4735–11 675)	7596 (6417)	5799 (3866–9213)	15	–24

IQR = interquartile range. SD = standard deviation.

**Figure 1.**
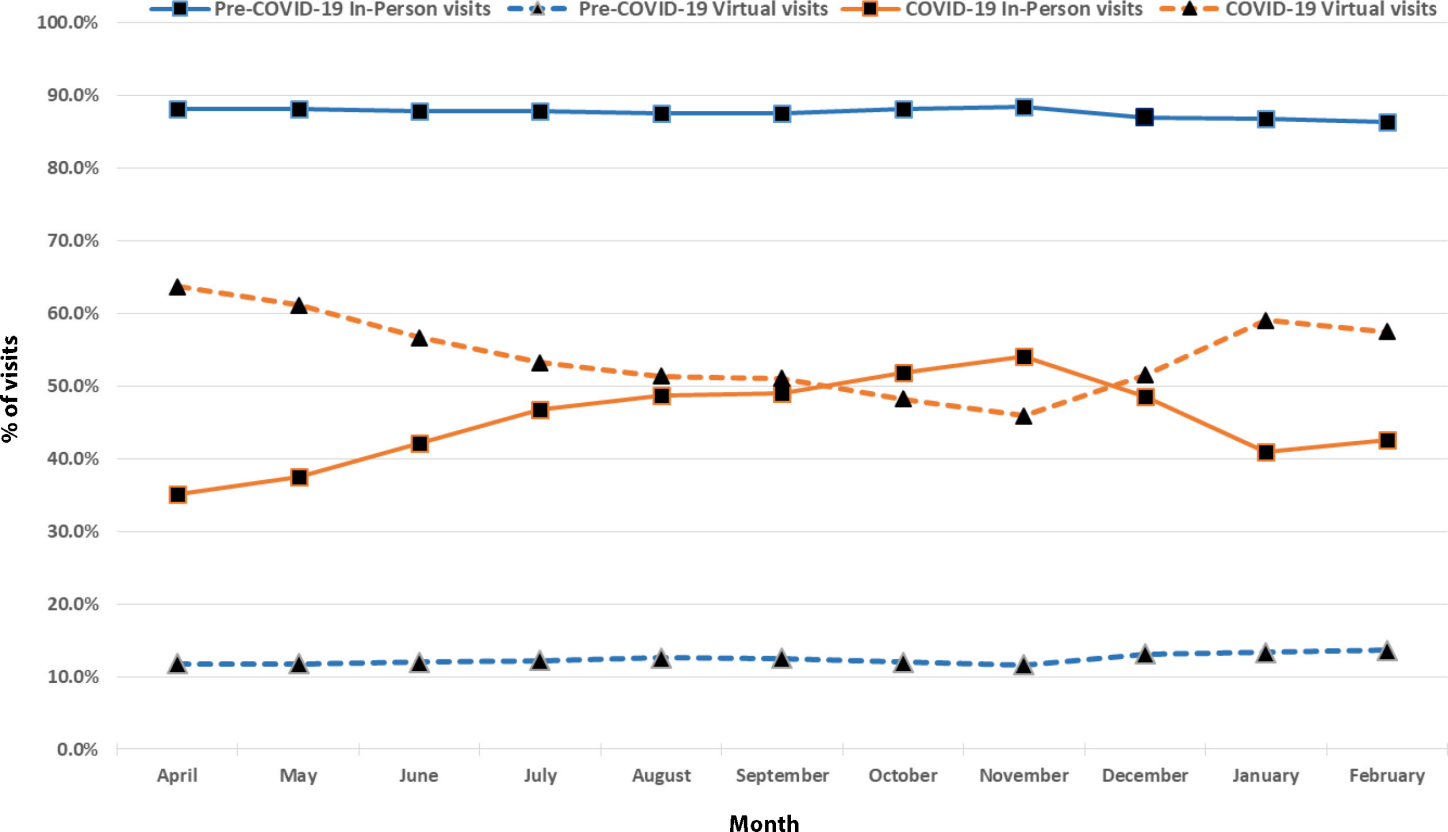
Monthly primary care visits by visit type (in-person, virtual) during April 2019–February 2020 (pre-COVID-19) and April 2020–February 2021 (COVID-19)

**Figure 2.**
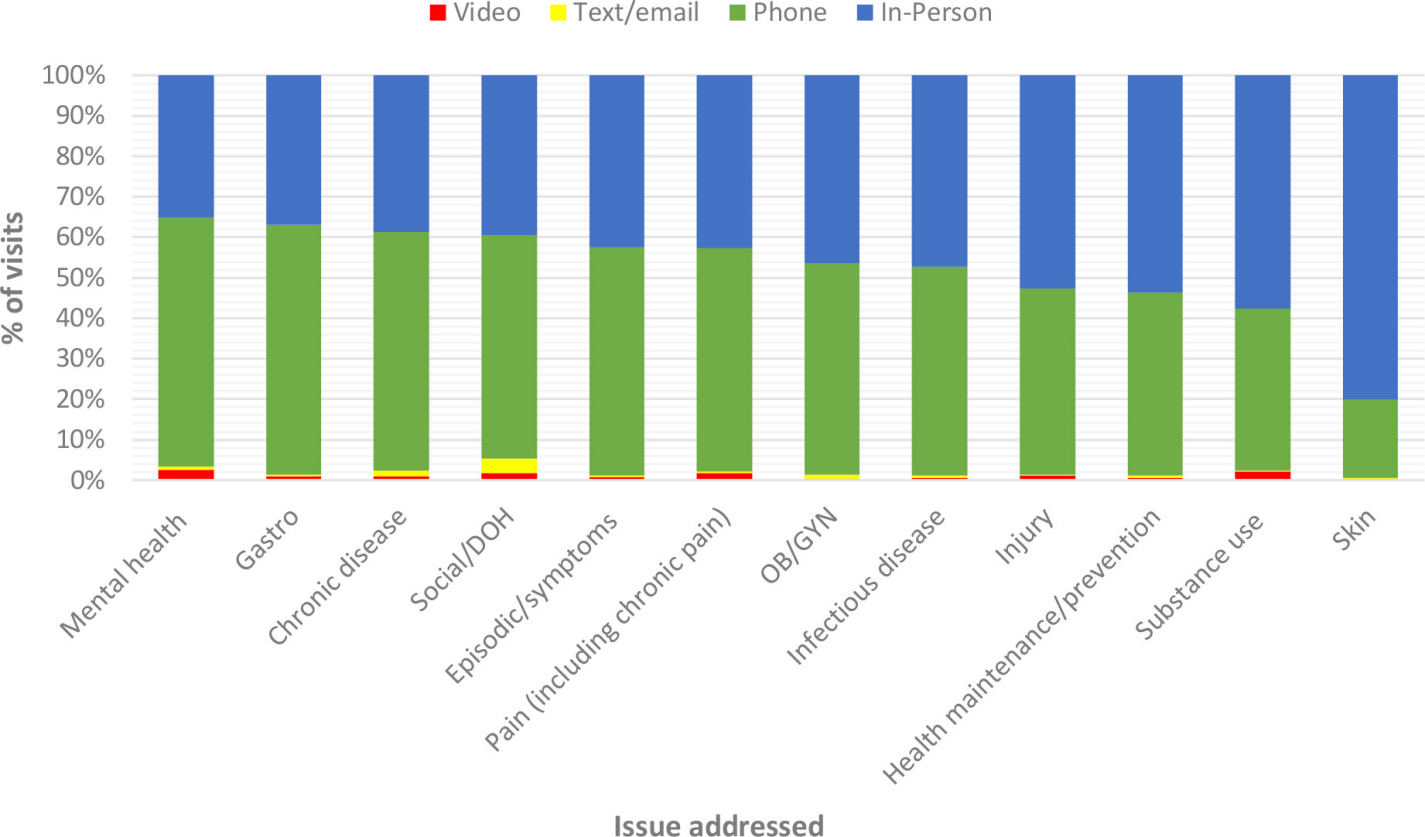
Issues addressed by visit modality during pandemic period, April 2020–February 2021. DOH = determinants of health. OB or GYN = obstetrics or gynaecology.

Before the pandemic, on average 88% of care was provided in-person (Figure 1). Over the course of the pandemic period, however, virtual visits made up, on average, 54% of visits and in-person visits declined by 48%. Telephone was the leading virtual modality used (96%), while video (2%) and text or email-based visits (2%) were rare. The latter two were most commonly used for issues related to mental health, determinants of health, and chronic disease (Figure 2). Video platforms used included Ontario Telemedicine Network, PS Suite EMR, Zoom, and social media (for health promotion programmes). Table 3 summarises the top 20 service types in which the greatest decreases and increases in service provision were observed. Drops in services ranged from 28%–82%, with the greatest declines seen in written patient care instructions (82%) and periodic health examinations (73%). The greatest increase in service provision was seen in services related to palliative care (212%) and individual counselling (85%).

**Table 3.** Total number of services provided during April 2019–February 2020 (pre-COVID-19) and April 2020–February 2021 (COVID-19)

Service type	Sum of service type providedPre-COVID-19 (*n* = 36), *n*	Sum of service type provided during COVID-19 (*n* = 36), *n*	% change
Written patient care instructions	3977	710	–82%
Periodic health examination	9736	2601	–73%
Well child health examination	3300	1506	–54%
Foot care	53 774	25 155	–53%
Accompaniment	7351	3768	–49%
Physical therapy	23 386	13 016	–44%
Forms completion	47 509	28 507	–40%
Preventive care	127 353	86 925	–32%
Interpretation services	18 783	13 541	–28%
Patient intake	42 629	30 879	–28%
Recommendation or assistance	104 309	125 904	21%
Postnatal care	1201	1464	22%
Health card registration	1593	1958	23%
Case management	65 805	81 043	23%
Care plan documentation	11 334	13 979	23%
Mental health care	34 160	42 207	24%
Breastfeeding counselling	2055	2653	29%
Occupational therapy	1304	1691	30%
Individual counselling	35 439	65 469	85%
Palliative care	350	1092	212%

Physicians, nurse practitioners, and nurses had a similar distribution of in-person to virtual visits, with a mean of 52% (standard deviation [SD] 0.05) of visits conducted virtually. Of virtual modalities, an average of 96% (SD 0.01) of visits were conducted over telephone, with only 2% (SD 0.005) through video and 1% (SD 0.004) conducted using text or email. In contrast, diabetes educators, dieticians, and counsellors conducted on average 77% (SD 0.07) of their visits through virtual delivery. Chiropodists, however, had on average 74% of visits conducted in-person (data not shown).

When evaluating the distribution of all modalities across patients‘ socioeconomic status during the pandemic time period, an almost even distribution of in-person and virtual visits was seen (see Table 4). There were no noticeable differences in access to the three virtual modalities across patient factors. In addition, urban and rural sites had very similar use of virtual and in-person care (data not shown).

**Table 4.** Patient characteristics by visit modality

Sociodemographic variable	*n* (%)			
	In-person	Telephone	Text or email	Video
Age, years				
0–12	18 652 (60.7)	11 271 (36.7)	518 (1.7)	263 (0.9)
13–26	21 591 (43.9)	25 078 (51.0)	1362 (2.8)	1154 (2.3)
27–40	36 415 (45.1)	41 230 (51.1)	1610 (2.0)	1465 (1.8)
41–60	58 937 (43.9)	71 449 (53.2)	2037 (1.5)	1871 (1.4)
61–75	51 762 (46.6)	57 129 (51.4)	946 (0.9)	1216 (1.1)
>76	28 708 (50.6)	27 180 (47.9)	347 (0.6)	508 (0.9)
Sex				
Female	127 216 (45.3)	145 205 (51.8)	4001 (1.4)	4106 (1.5)
Male	84 591 (49.0)	83 845 (48.6)	2161 (1.3)	2031 (1.2)
Other	4258 (44.9)	4287 (45.2)	658 (6.9)	280 (3.0)
Education				
High school or less	74 366 (47.1)	80 263 (50.8)	1620 (1.0)	1598 (1.0)
Post-secondary or equivalent	49 297 (44.0)	59 932 (53.5)	1120 (1.0)	1659 (1.5)
Other	92 402 (47.9)	93 142 (48.3)	4080 (2.1)	3160 (1.6)
Income				
$0–$14 999	34 070 (46.3)	37 835 (51.4)	929 (1.3)	817 (1.1)
$15 000–$19 999	11 606 (46.3)	12 903 (51.5)	271 (1.1)	288 (1.1)
$20 000–$24 999	8687 (45.7)	9886 (52.0)	225 (1.2)	198 (1.0)
$25 000–$29 999	5966 (46.4)	6629 (51.6)	99 (0.8)	153 (1.2)
$30 000–$34 999	6137 (46.4)	6835 (51.7)	120 (0.9)	135 (1.0)
$35 000–$39 999	4965 (45.4)	5795 (53.0)	70 (0.6)	99 (0.9)
$40 000–$59 999	12 029 (45.4)	13 817 (52.2)	260 (1.0)	375 (1.4)
>$60 000	18 487 (44.6)	21 865 (52.8)	374 (0.9)	699 (1.7)
Other	114 118 (47.5)	117 772 (49.1)	4472 (1.9)	3653 (1.5)
Spoken language				
English	157 037 (45.7)	177 566 (51.7)	4705 (1.4)	4461 (1.3)
French	6837 (47.8)	7094 (49.6)	148 (1.0)	222 (1.6)
Other	52 191 (49.9)	48 677 (46.6)	1967 (1.9)	1734 (1.7)
Household composition				
Living with others	94 027 (46.0)	106 255 (51.9)	1924 (0.9)	2420 (1.2)
Living alone	28 243 (46.9)	30 634 (50.9)	630 (1.0)	702 (1.2)
Other	93 795 (47.4)	96 448 (48.8)	4266 (2.2)	3295 (1.7)
**Overall**	**216 065** (**46.7**)	**233 337** (**50.4**)	**6820** (**1.5**)	**6417** (**1.4**)

## Discussion

### Summary

Over the course of the pandemic period, CHCs experienced a moderate decline in visits made, patients seen, issues addressed, and meaningful changes in the types of services provided. Care delivered virtually was primarily done through telephone visits, with very little use of video or text or email-based visits. The distribution of virtual modalities across providers varied according to the provider type with interprofessional team members (aside from chiropodists) providing a greater proportion of their care virtually. Chiropodists, owing to the nature of their profession, had the greatest proportion of in-person visits. Access to in-person and virtual care did not vary across patient characteristics suggesting equitable access to all modalities of care.

### Strengths and limitations

This study’s strengths include the following: extraction of data from EMR; access to data on visit modalities for issues addressed, services provided, and provider types including interprofressional team members; and sociodemographic data from patient populations who often face multiple barriers to accessing care. The use of multiple modalities in a single visit were unable to be reported; visit type may have been misclassified in some cases as the EMR template defaults to ‘in-person‘ unless the provider changed it to another type.

### Comparison with existing literature

Despite CHCs offering little virtual care before the pandemic, over half of centres felt prepared in shifting towards greater use of virtual modalities owing to the COVID-19 pandemic. This may in part be owing to CHCs employing a salary-funded model that, unlike other models, would not have been influenced by the availability of billing codes.^2,5–17,17^

Knowing that various determinants of health can affect access to digital technology and digital literacy,^18,19^ the CHCs’ emphasis in maintaining in-person care may have prevented a greater decrease in overall visits compared with other primary care settings. For example, according to a systematic review, the median reduction in healthcare visits was 42%, nearly double the decline observed in CHCs.^14^ Furthermore, a Canadian study looking at physician billing data reported a mean reduction of 28% for total visits, far greater than the 11% seen in the present study.^13^ The study also observed a greater decline of in-person visits (that is, 79% versus 48% in the present study) and a higher average of virtual visits (that is, 71% in the present study versus 54%).^13^

In the present study, the limited use of video and text or email-based visits was expected, as concerns about patient access to Wi-Fi and digital literacy were reported by almost half of participating CHCs. A survey conducted by Canada Health Infoway, similarly reported telephone visits as the main virtual modality.^20^ Between April and August 2020, approximately 76% of virtual visits in Ontario were conducted over telephone, 20% over video, and 5% through secure messaging. Other provinces also reported similar distributions.^20^

The study’s data demonstrated large increases in palliative care and mental health services. This rise in palliative care may have been a result of COVID-19 restrictions further restricting access to an already limited service,^21^ and CHC providers stepping in to address this increased demand. As the pandemic has also exacerbated issues related to mental health,^22^ it is no surprise that CHCs responded to the needs of their patients by increasing these supports by 85%.

Despite concerns that patients who were poorer or had other barriers to access would be disadvantaged by the shift to virtual care, visit modalities did not vary across any patient characteristics. The even distribution among telephone versus in-person visits could be a result of CHC’s recognising the impact of limiting in-person visits, as well as inequitable access to virtual care earlier on, and procuring telephones and data plans to patients in order to maintain their access to care.^23^ Greater use of video visits among those with higher education and household income had also been anticipated as internet access and digital literacy is influenced by socioeconomic factors.^24^ However, since the use of telephone was the primary source of virtual visits, this may have resulted in other barriers not being identified.^25^ Overall, the study results point to a rapid, equitable shift to virtual care while maintaining in-person care when needed. In-person service delivery was ongoing and the shift in types of service demonstrated a responsiveness to the emerging needs of the patients.

Relatedly, the data did not demonstrate any considerable differences owing to rurality with respect to both changes in primary care services and distribution of visit modalities. The even distribution of video visits was especially unexpected, given how unequal access to high-speed internet is across the country. The Canadian Radio-television and Telecommunication Commission (CRTC) has highlighted a significant digital equity gap in regard to rural households accessing reliable and affordable high-speed internet.^26^ It reports that only 37% of rural households in 2017 had adequate internet speeds, including those required for telehealth services, in comparison with 97% of urban homes.^26^ Within this context, a greater use of video-based visits among urban centres was expected to be seen.

### Implications for research and practice

The results of this study describe the challenges experienced by CHCs during the rapid transition to virtual care, as well as impacts on primary care service delivery. The results demonstrate a large shift towards virtual delivery while maintaining in-person care and increases in specific services presumably as a response to patients’ needs. No meaningful differences were found in virtual versus in-person care related to patient characteristics or rurality of centres. Future studies are needed to explore the distribution of modalities for different types of care and services in other primary care settings, as well as how to best select the most appropriate modality for patients and service types.
